# USING PHOTOVOICE TO GAIN A DEEPER UNDERSTANDING OF OLDER ADULTS EXPERIENCE OF CLIMATE CHANGE

**DOI:** 10.1093/geroni/igae098.1009

**Published:** 2024-12-31

**Authors:** Hui Zhao, Anne Stewart, Christine Fasching Maphis, Francesca Ezeokonkwo, Jennifer Nelson-Faulconer, Tiffany Tanaka, Nehemiah Winesberry

**Affiliations:** James Madison University, Harrisonburg, Virginia, United States; James Madison University, Harrisonburg, Virginia, United States; Sentara Community Care, Harrisonburg, Virginia, United States; Holy Family University, Philadelphia, Pennsylvania, United States; James Madison University, Harrisonburg, Virginia, United States; James Madison University, Harrisonburg, Virginia, United States; James Madison University, Harrisonburg, Virginia, United States

## Abstract

Older adults are disproportionately impacted by the climate crisis. However, their concerns and how they perceive and respond to climate change, in their own words, remain unclear. We conducted a qualitative study employing an innovative autophotography process with semi-structured interviews to capture insights into older adults’ knowledge and beliefs regarding climate change issues. Constant comparison was used to analyze data. Seventeen community-based older adults living in Virginia Shenandoah Valley region were recruited and 14 of them completed the study. Participants were 67–85 years old white adults, with the majority holding a graduate degree. From the preliminary data analysis, four themes emerged around exploring older adults’ experiences and perceptions of climate change and each theme included sub-themes: 1) Perceived environmental issues impacting on health and life. Participants identified various specific concerns and priorities that older adults have regarding climate change, such as impacts on health or quality of life. 2) Barriers. Several factors hampered the effective addressing of climate change issues, including limited awareness, fixed mindsets, a lack of intergenerational conversation, and financial constraints. 3) Resilience and engagement. Older adults utilized resilience-building strategies that helped them cope with climate change-related challenges and empowered them to advocate for community resilience efforts. Our findings underscore the importance of further education about climate-associated risks. Including older adults in the conversation about climate change is crucial for promoting intergenerational dialogue and fostering mutual understanding and collaboration across age groups. Policy changes can facilitate effective actions on climate change-related issues.

